# Novel regulation of renal gluconeogenesis by Atp6ap2 in response to high fat diet via PGC1-α/AKT-1 pathway

**DOI:** 10.1038/s41598-021-90952-7

**Published:** 2021-05-31

**Authors:** Safia Akhtar, Silas A. Culver, Helmy M. Siragy

**Affiliations:** Department of Medicine, University of Virginia Health System, P.O. Box 801409, Charlottesville, VA 22903 USA

**Keywords:** Kidney, Kidney diseases

## Abstract

Recent studies suggested that renal gluconeogenesis is substantially stimulated in the kidney in presence of obesity. However, the mechanisms responsible for such stimulation are not well understood. Recently, our laboratory demonstrated that mice fed high fat diet (HFD) exhibited increase in renal Atp6ap2 [also known as (Pro)renin receptor] expression. We hypothesized that HFD upregulates renal gluconeogenesis via Atp6ap2-PGC-1α and AKT pathway. Using real-time polymerase chain reaction, western blot analysis and immunostaining, we evaluated renal expression of the Atp6ap2 and renal gluconeogenic enzymes, PEPCK and G6Pase, in wild type and inducible nephron specific Atp6ap2 knockout mice fed normal diet (ND, 12 kcal% fat) or a high-fat diet (HFD, 45 kcal% fat) for 8 weeks. Compared with ND, HFD mice had significantly higher body weight (23%) (*P* < 0.05), renal mRNA and protein expression of Atp6ap2 (39 and 35%), PEPCK (44 and 125%) and G6Pase (39 and 44%) respectively. In addition, compared to ND, HFD mice had increased renal protein expression of PGC-1α by 32% (*P* < 0.05) and downregulated AKT by 33% (*P* < 0.05) respectively in renal cortex. Atp6ap2-KO abrogated these changes in the mice fed HFD. In conclusion, we identified novel regulation of renal gluconeogenesis by Atp6ap2 in response to high fat diet via PGC1-α/AKT-1 pathway.

## Introduction

The kidney plays a pivotal role in systemic glucose homeostasis by regulation of glucose reabsorption, glycolysis, and gluconeogenesis. Previous studies demonstrated the role of the renal proximal tubules in gluconeogenesis^1^. Liver and kidney are the only two organs capable of making glucose because of the presence of two rate-limiting enzymes for glucose production, phosphoenolpyruvate carboxykinase (PEPCK) and glucose-6-phosphatase (G6Pase). It was generally believed that the liver was only the source of glucose except in acidotic conditions and after prolonged fasting. After years of research in vivo and in vitro there are ample evidence that the kidney can make glucose and release it under various conditions. In individuals undergoing liver transplantation, circulating glucose level does not drop to zero after removal of the liver^2^. The increased renal fractional contribution of gluconeogenesis to post-absorptive endogenous glucose production in type 2 diabetes is well established^3^. Similarly, a number of studies have demonstrated increased renal gluconeogenesis in obesity^1,4^, but the involved mechanisms are less clear. In the kidney, gluconeogenesis occurs in the cells of the proximal tubule^5,6^. Much of the glucose produced in the kidney is used by the renal medulla, while the role of the renal gluconeogenesis in maintaining blood glucose levels becomes more important during prolonged fasting and liver failure. Unlike the liver, the kidney has no significant glycogen stores and contributes to the maintenance of blood glucose homeostasis only through gluconeogenesis and not through glycogenolysis^7,8^.

The insulin signalling cascade involves several major pathways such as the PI3K/Akt pathway and the MAPK/MEK pathway. Impairment in the PI3K/AKT pathway is associated with glucose intolerance and insulin resistance along with increased expression of gluconeogenic enzymes^9,10^ Hence, enhancement of the PI3K/AKT activities may be a promising strategy for improving glucose tolerance and insulin sensitivity. The best-characterized pathway for transcriptional control of gluconeogenic gene expression involves PGC1-α, which plays a pivotal role as a coactivator of FOXO1^11–13^.

The Atp6ap2 (also known as (pro)renin receptor) is localized in various kidney structures, including proximal and distal tubules and collecting ducts. Recently, we reported that renal Atp6ap2 expression is upregulated in high-fat diet and in diabetes^14–16^. Our previous studies also showed the Atp6ap2 role in regulating AKT-1 in streptozotocin (STZ) induced type-1 diabetes^17,18^. Although the kidney has been known to play an important role in systemic glucose homeostasis, the mechanisms involved in the regulation of gluconeogenesis in the kidney remain poorly understood. Recently, it was reported that gluconeogenesis is dually regulated in the kidney by both the reabsorbed glucose via the SGLTs in the luminal membrane and insulin signaling transduced from the basolateral membrane in the proximal tubular cells^19,20^. Based on previous observations and lack of clear understanding, our study aimed to determine the relationship of Atp6ap2 and renal gluconeogenesis in normal and high-fat diet (HFD)-induced obesity in mice. We tested the hypothesis that HFD upregulates renal gluconeogenesis via Atp6ap2-PGC-1α and AKT signaling cascade.

## Results

### Body weight and random blood glucose measurement

**Figure 1:**
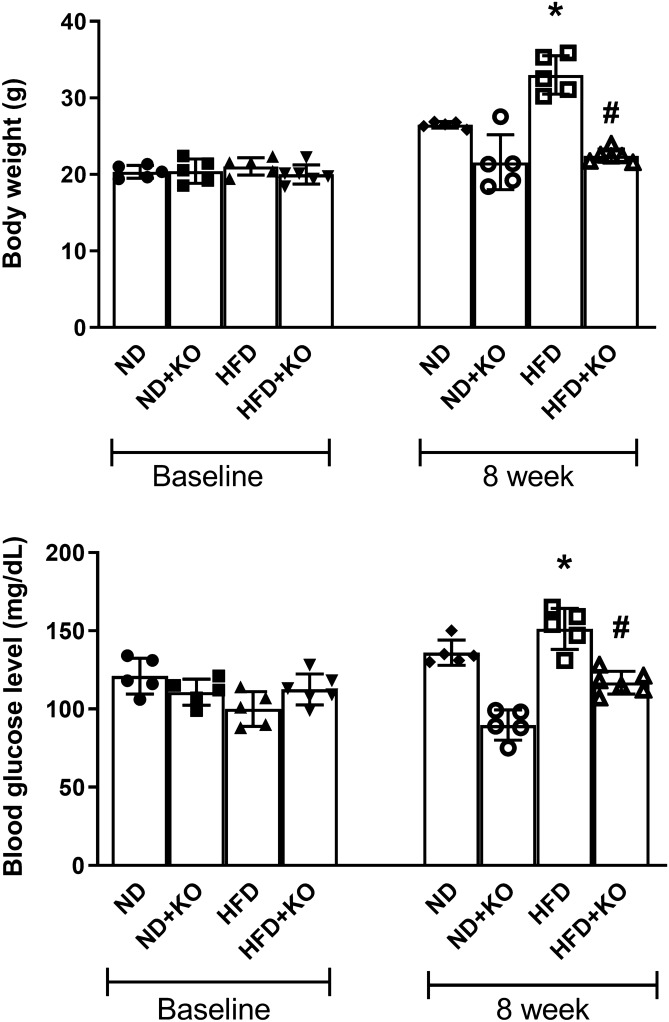
Body weight and plasma glucose. Body weight and plasma glucose level after 8 weeks following normal diet (ND) and high fat diet (HFD) in mice with or without Atp6ap2-KO. Data presented as mean ± SEM, *P < 0.05 versus HFD; ^#^*P* < 0.05 versus HFD + KO, n = 5–6 each group.

At baseline, there were no significant differences in body weight or blood glucose between all animal groups. Compared to normal diet (ND) control mice, HFD fed mice had a significant (*P* < 0.05) increase in body weight and random blood glucose by 23% and 15%. The body weight and blood glucose of inducible nephron specific Atp6ap2 KO mice receiving HFD (HFD + KO) was significantly lower by 31% and 24% respectively than HFD alone mice (*P* < 0.05) (Fig. 1).

### Effect of HFD on Atp6ap2 mRNA and protein expression

**Figure 2:**
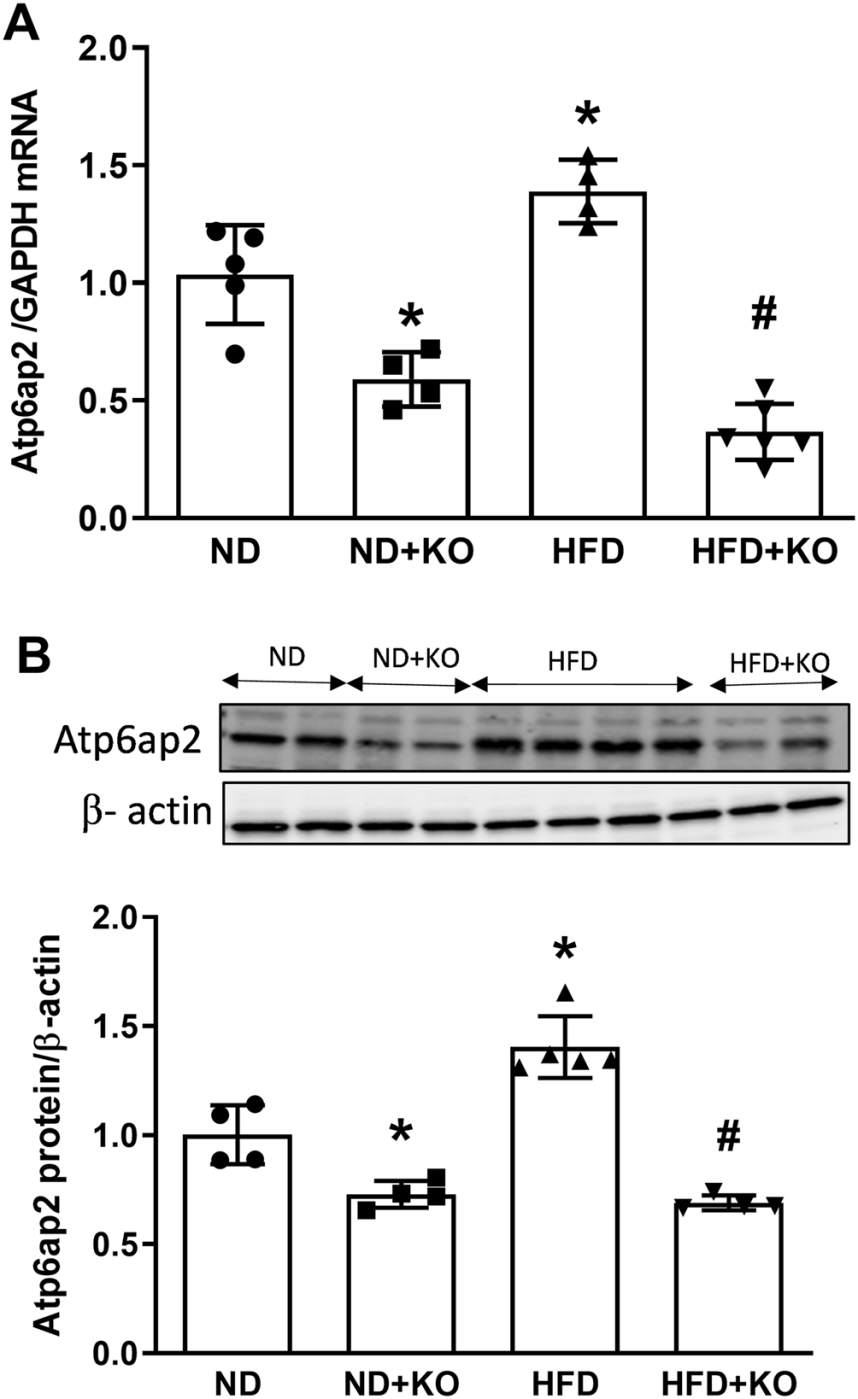
Renal cortical expression of Atp6ap2. Renal cortical mRNA and protein expression of Atp6ap2 after 8 weeks following normal diet (ND) and high fat diet (HFD) in mice with or without Atp6ap2-KO. Data presented as mean ± SEM, *P < 0.05 versus HFD; ^#^*P* < 0.05 versus HFD + KO, n = 5–6 each group.

**Figure 3:**
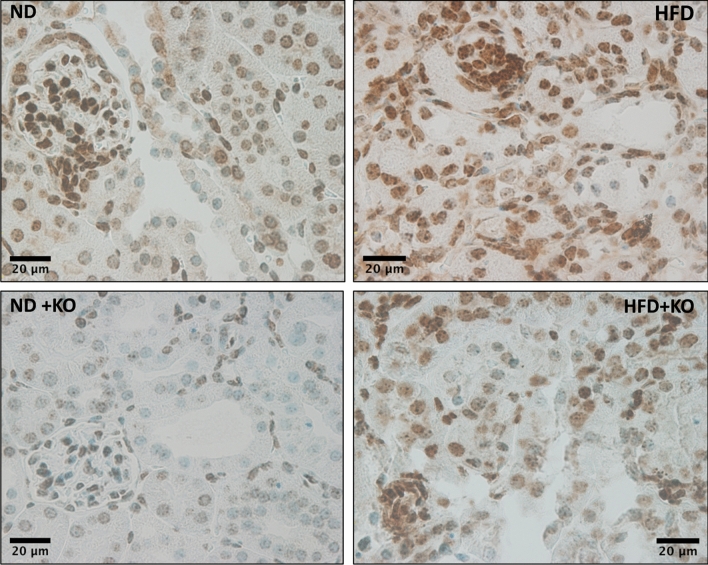
Immunostaining of renal Atp6ap2. Immunostaining of renal cortical Atp6ap2 after 8 weeks following normal diet (ND) and high fat diet (HFD) in mice with or without Atp6ap2-KO.

Compared to ND control mice, renal cortex mRNA and protein expression of Atp6ap2 was significantly increased by 39 and 35% (*P* < 0.05) respectively in the HFD mice. Whereas Atp6ap2 KO mice consuming ND (ND + KO) had significant decrease in Atp6ap2 mRNA and protein expression by 60 and 72% respectively (Fig. 2) when compared to ND group. Similarly, compared to HFD, HFD + KO had significant decrease in Atp6ap2 mRNA and protein expression by 71% and 75% (*P* < 0.05), respectively (Fig. 2). HFD fed mice had significant increase in renal Atp6ap2 immunostaining than ND mice. Compared to ND and HFD mice groups, ND + KO and HFD + KO had significant decrease in the immunostaining of Atp6ap2 in renal cortex (Fig. 3).

### Effect of Atp6ap2 on PEPCK, G6Pase and FBpase expression in HFD kidneys

**Figure 4:**
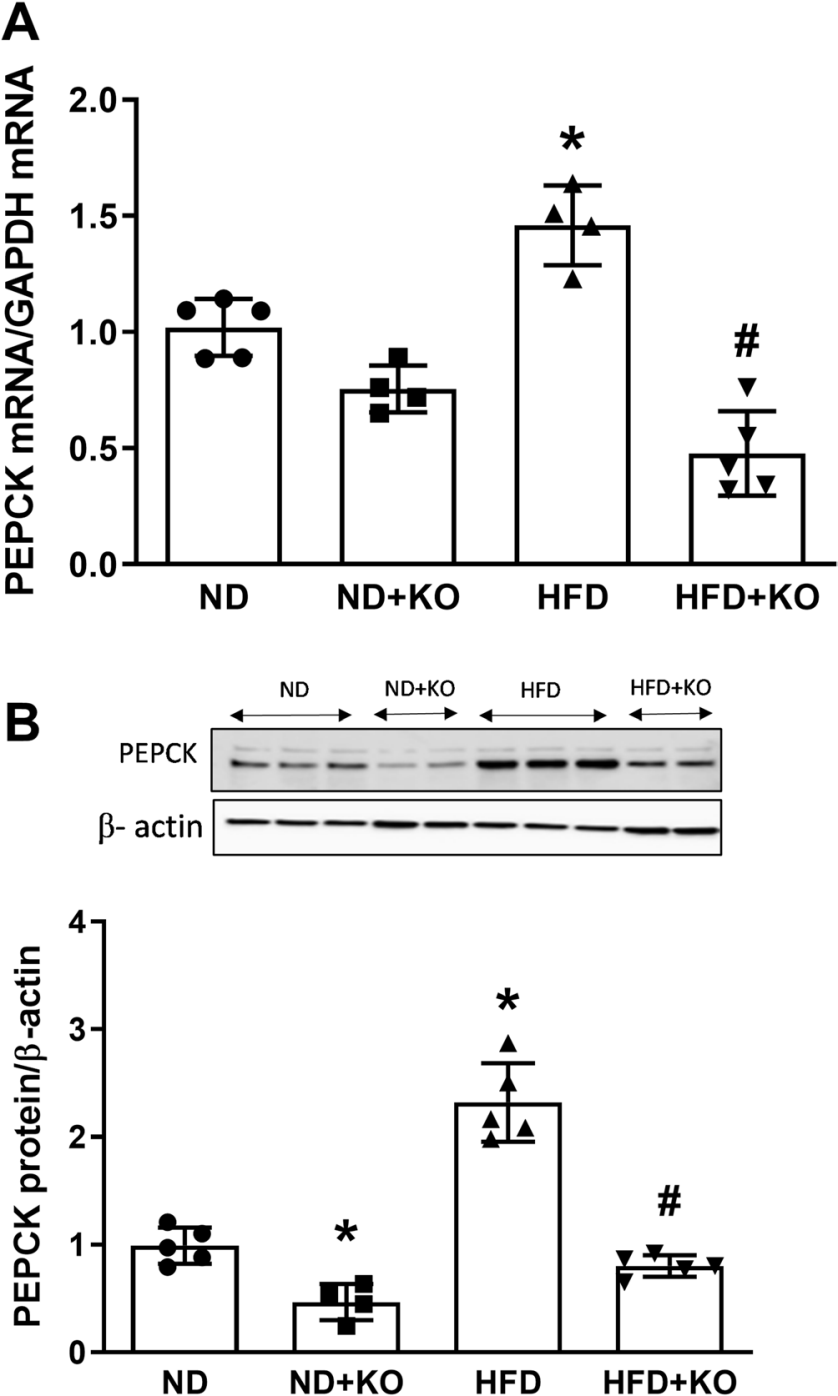
Renal cortical expression of PEPCK. Renal cortical mRNA and protein expression of PEPCK after 8 weeks following normal diet (ND) and high fat diet (HFD) in mice with or without Atp6ap2-KO. Data presented as mean ± SEM, *P < 0.05 versus HFD; ^#^*P* < 0.05 versus HFD + KO, n = 4–5 each group.

**Figure 5:**
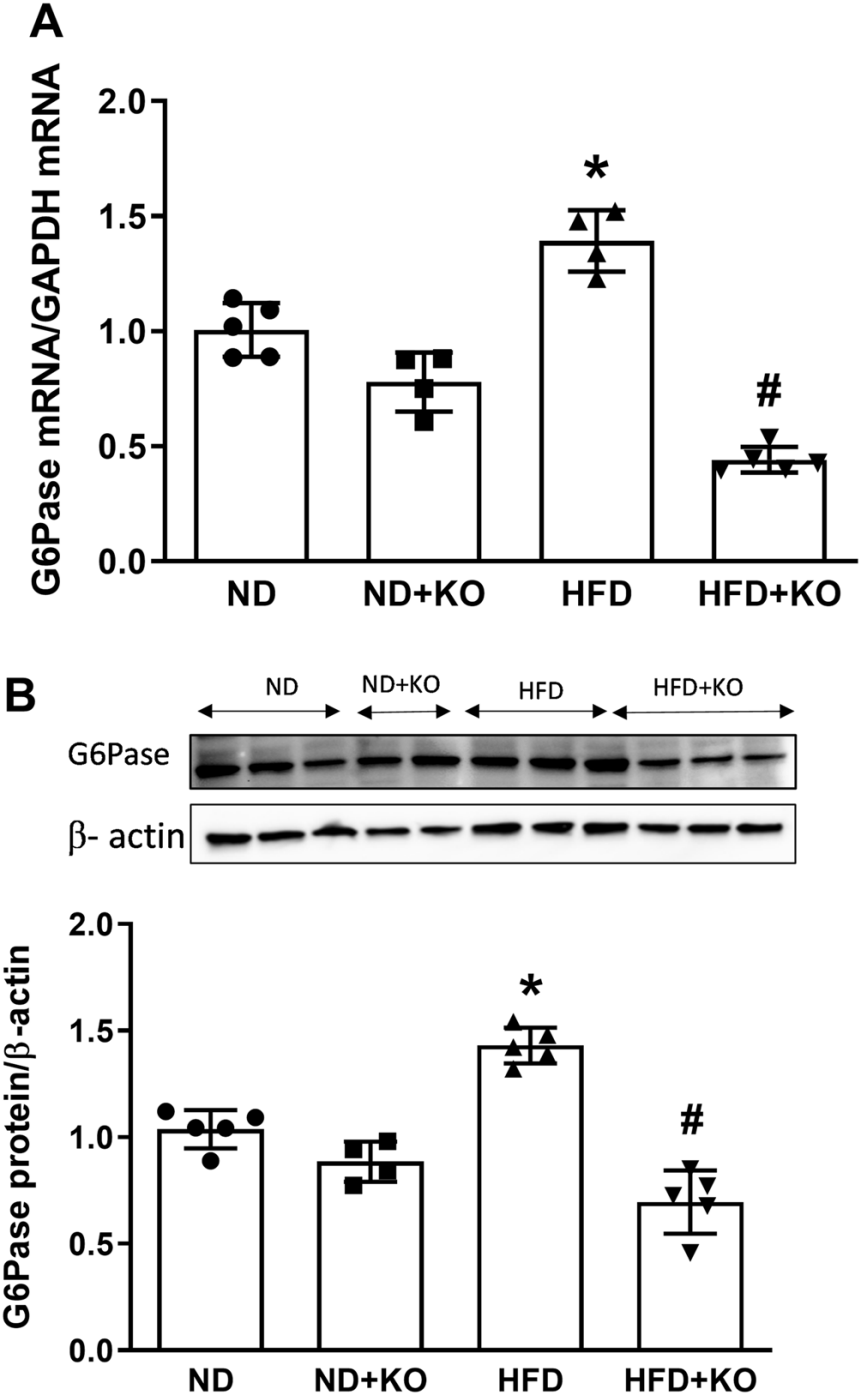
Renal cortical expression of G6Pase. Renal cortical mRNA and protein expression of G6Pase after 8 weeks following normal diet (ND) and high fat diet (HFD) in mice with or without Atp6ap2-KO. Data presented as mean ± SEM, *P < 0.05 versus HFD; ^#^*P* < 0.05 versus HFD + KO, n = 4–5 each group.

**Figure 6:**
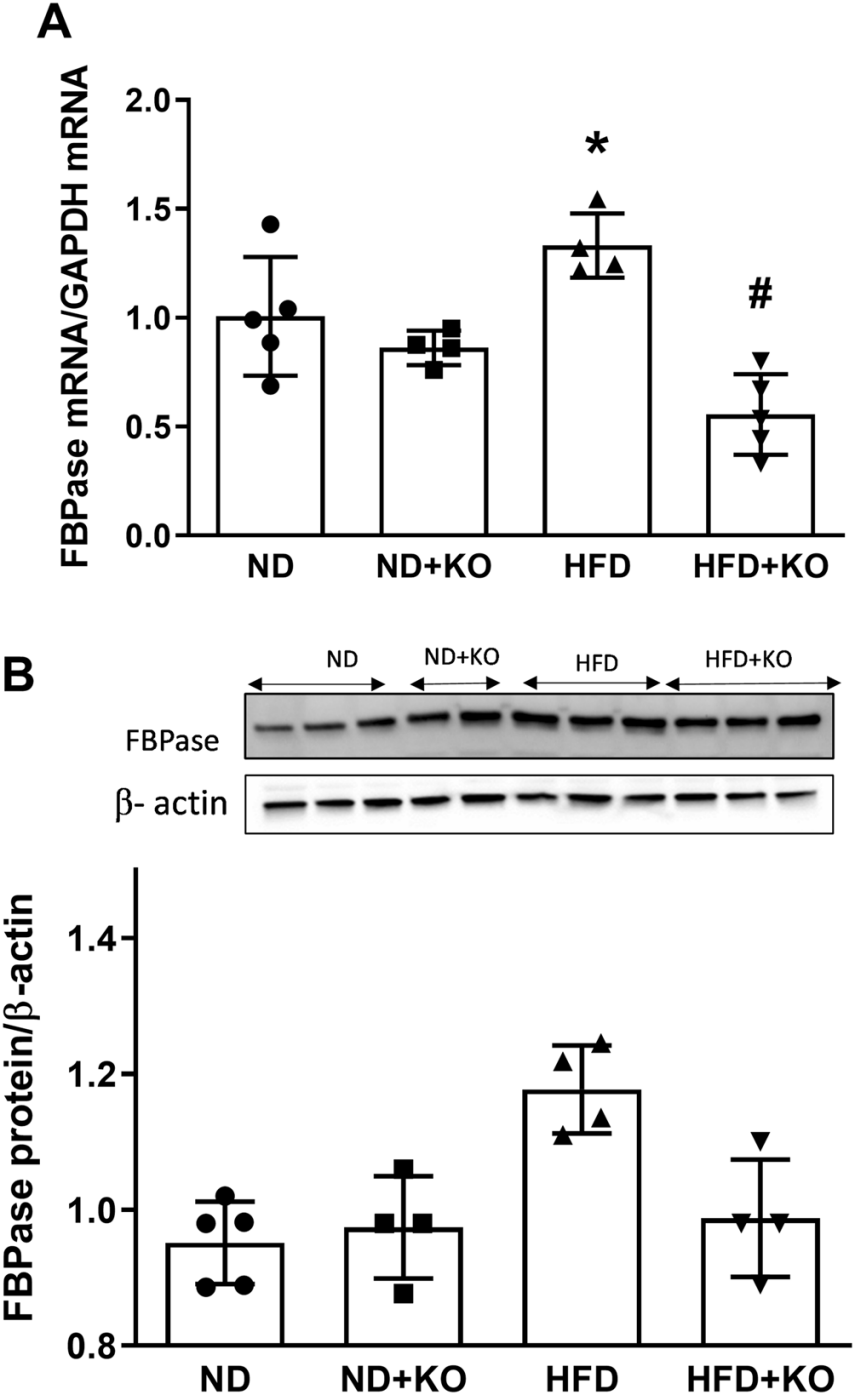
Renal cortical expression of FBPase. Renal cortical mRNA and protein expression of FBPase after 8 weeks following normal diet (ND) and high fat diet (HFD) in mice with or without Atp6ap2-KO. Data presented as mean ± SEM. *P < 0.05 versus HFD; ^#^*P* < 0.05 versus HFD + KO, n = 4–5 each group.

The gluconeogenic enzymes pyruvate carboxylase, PEPCK, fructose 1, 6-bisphosphatase (FBPase), and G6Pase are present in the kidney and contribute to renal gluconeogenesis. In humans, gluconeogenesis takes place primarily in the liver and, to a lesser extent, the renal cortex. We evaluated the renal gluconeogenic enzymes to determine whether gluconeogenesis is enhanced during HFD intake and whether it is dependent on Atp6ap2. Compared to ND mice, HFD mice had significant increase in mRNA and protein expression of PEPCK by 44 and 125% (Fig. 4) and G6Pase (Fig. 5) by 39 and 44% (*P* < 0.05) respectively. Compared to ND mice, HFD alone significantly increased mRNA levels of FBPase (Fig. 6) by 33% (*P* < 0.05). HFD group had slight increase in FBPase protein expression but was not statistically different from the ND group (Fig. 6). Compared to HFD alone, there were significant decreases in mRNA and protein levels of PEPCK by 65 and 61% respectively (*P* < 0.05) (Fig. 4), and mRNA level of G6Pase (Fig. 5) and FBPase (Fig. 6) by 67 and 60% (*P* < 0.05) in HFD + KO mice. HFD + KO group had slight decrease in FBPase protein expression but was not statistically different from the HFD group (Fig. 6).

### Effect of Atp6ap2 on PGC-1α and p/t-AKT-1 expression in HFD kidneys

**Figure 7:**
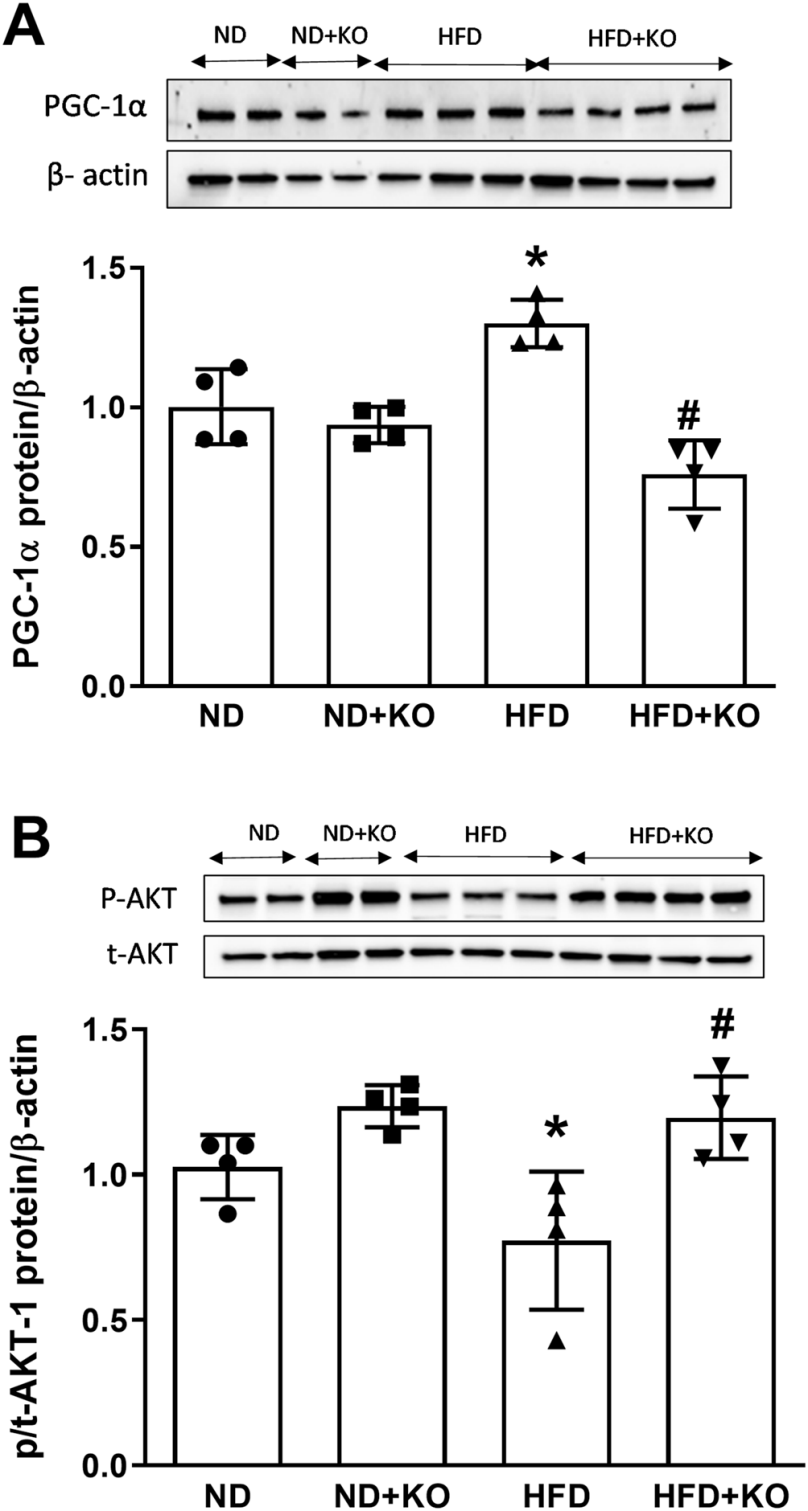
Renal cortical expression of PGC-1α and p/t-AKT-1. Renal cortical protein expression of PGC-1 α and p/t-AKT-1 after 8 weeks following normal diet (ND) and high fat diet (HFD) in mice with or without Atp6ap2-KO. Data presented as mean ± SEM, *P < 0.05 versus HFD; ^#^*P* < 0.05 versus HFD + KO, n = 3–5 each group.

Compared to ND mice, there were significant increases in protein expression of PGC-1α by 32% (*P* < 0.05) and decreases in p/t-AKT-1 protein expression by 33% (*P* < 0.05) in the renal cortex of mice fed HFD (Fig. 7). HFD + Atp6ap2-KO significantly decreased protein expression of PGC-1α by 77% (*P* < 0.05), and increases p/t-AKT-1 by 22% respectively (*P* < 0.05) (Fig. 7). Compared to ND control mice, there were no differences in the protein expressions of PGC-1α and p/t-AKT-1 in the renal cortex of Atp6ap2 KO mice consuming ND (Fig. 7).

## Discussion

In the present study, we aimed to evaluate the role of Atp6ap2 in renal gluconeogenesis during high-fat diet intake in mice. Our first finding demonstrated that HFD significantly upregulated Atp6ap2 mRNA and protein expression in the mice renal cortex. This finding is in agreement with the previous observation that renal Atp6ap2 expression is upregulated in the kidneys of high-fat fed mice^16^. We also observed HFD significantly increased body weight, which was abrogated in nephron specific Atp6ap2KO mice. The cause of low body weight in Atp6ap2KO mice is not clear and could be, in part, due to increased urine volume (data not shown). Previous studies reported regulation of renal aquaporin-2 expression and activity by Atp6ap2. Inducible nephron specific Atp6ap2KO was associated with decreased aquaporin-2 expression, increased urine volume and reduction in body weight^16,21^. Thus, increased urine volume in Atp6ap2KO mice could partially explain the observed low body weight in the KO groups. Then, we demonstrated the novel findings that renal gluconeogenic enzymes PEPCK and G6Pase were significantly increased in response to HFD. PEPCK is a key enzyme in the formation of gluconeogenesis in rodent liver and kidney cortex^22^. Another novel finding that we found was knockdown of the renal tubular cells Atp6ap2 attenuated gluconeogenesis in the kidney in response to HFD. To our knowledge, this is the first report of regulation of renal gluconeogenesis by Atp6ap2 in response to HFD. Next, our study demonstrated that knockdown of Atp6ap2 in the renal tubular cells attenuated the PGC-1α and enhanced AKT signaling pathway. These results clearly demonstrated the intricate and direct interaction between Atp6ap2 and renal gluconeogenesis in HFD fed mouse model via PGC1-α/ AKT-1 pathway.

Gluconeogenesis occurs predominately in the liver and kidney and is essential for the production of glucose during extended fasting when glycogen stores have been depleted. Unlike type-2 diabetic patients, very limited studies on animal models of obesity have been reported until now to display an increased renal gluconeogenesis. Previous studies reported that renal gluconeogenesis was substantially stimulated in patients with type 2 diabetes and in the Zucker diabetic fatty rat and potentially could contribute to the observed hyperglycemia in this population^1^. In Zucker diabetic fatty rat, circulating glucocorticoids and cAMP demonstrated to play a substantial role by augmenting the expression of key gluconeogenic genes via upregulation of the expression of the phosphoenolpyruvate carboxykinase gene and the enzymatic activities of phosphoenolpyruvate carboxykinase and glucose-6-phosphatase^1^. Interestingly, several factors were reported to be involved in the long-term stimulation of renal gluconeogenesis such as metabolic acidosis and increased circulating catecholamines and exert a direct effect on renal glucose release^23–25^. However, these factors have systemic effects and may influence both hepatic and renal glucose release by increasing availability of gluconeogenic substrates and suppressing insulin secretion. Other factors like cortisol were reported to have long-term stimulatory influences on hepatic glucose release but their effects on renal gluconeogenesis are not well studied. Increased cortisol secretion has been attributed in android obesity^26^. Interestingly, small increases in serum cortisol may contribute to the abnormal glucose metabolism as was seen in the metabolic syndrome^27^. Both glucose and insulin may also influence renal gluconeogenesis. A study utilizing HK-2 cells, a model for renal proximal tubular cells, reported that high glucose levels suppressed the gluconeogenic enzyme PEPCK expression, which was reversed by treatment with a SGLT-2 inhibitor^28^. Similar studies explored the molecular mechanisms underlying the regulation of the gluconeogenic gene expression and documented its regulation by insulin and glucose^28^.

In the liver, chronic HFD is believed to contribute to gluconeogenesis via increased insulin resistance^29,30^. However, the current knowledge of the regulation of renal gluconeogenesis by HFD is not well elucidated. An obstacle in studying this field is the lack of an appropriate model that could evaluate the kidney contribution to gluconeogenesis during intake of HFD. Therefore, our study evaluated whether renal gluconeogenesis is stimulated by HFD and possible involvement of Atp6ap2 in this process. Our study confirmed that HFD enhances renal gluconeogenesis via increased Atp6ap2 expression. Several mechanisms seem to be involved in this process. One potential mechanism is that HFD-induced insulin resistance, which is known to modulate gluconeogenesis in the liver and kidney^31,32^. In addition, there is considerable evidence that HFD is associated with increased free fatty acid oxidation that in turn induces insulin resistance independent of visceral obesity and lead to increased gluconeogenesis^33^. Our results clearly confirmed the significant role of Atp6ap2 in regulating renal gluconeogenesis. This is to note that renal gluconeogenesis occurs only in renal proximal tubules because the expression of the rate-limiting enzymes PEPCK/PCK1 are restricted to segments S1, S2 and S3 of the proximal tubule in mice. Even under maximal stimulation, distal nephron segments are unable to perform gluconeogenesis which confirms that gluconeogenesis occurs only in proximal tubule cells and not in the latter portion of the nephron^34^.

Although the kidney has been known to play an important role in systemic glucose homeostasis, the mechanisms involved in the regulation of gluconeogenesis in the proximal tubule cells remains poorly understood. Recent study by Sasaki et al. demonstrated regulation of gluconeogenesis in the kidney by both the reabsorbed glucose via the SGLTs in the luminal membrane and insulin signaling transduced from the basolateral membrane in the proximal tubule cells^30^. They suggested that gluconeogenesis could be collaboratively regulated by both insulin signaling and the reabsorbed glucose in the proximal tubule cells. They also suggested that both insulin signaling and glucose reabsorption suppress the gluconeogenic genes expression by inactivation of FoxO-1 and PGC1α, respectively in the HK-2 cells. Another piece of evidence reported that insulin signaling is impaired in the obese mice. In a previous study, insulin significantly increased phosphorylation of AKT, insulin receptor substrate 1 and insulin receptor-β in lean mice lung tissue and isolated bronchi, and these effects were impaired in obese mice^35^. The current study provide evidence for involvement of Atp6ap2 in modulating some of the renal gluconeogenic signals/transcription factors in the HFD fed mice. We found that upregulation of renal Atp6ap2 activated the PGC-1α and down-regulated the AKT signaling pathway suggesting increased renal gluconeogenesis. In support of these data, specific deletion of the nephron Atp6ap2 activated AKT and attenuated PGC1-α suggesting potential role of Atp6ap2 in modulating renal gluconeogenesis. The current study has some limitations which we did not address. It is possible that increased expression of Atp6ap2 in the HFD kidney could have contributed to increased gluconeogenesis directly via AKT and PGC-1-α pathway, or indirectly by controlling insulin receptor signaling. Further studies should be done to evaluate the role of Atp6ap2 on insulin receptor and its signaling pathways.

Overall, our studies provide insight into a novel mechanism underlying the regulation of gluconeogenesis in the kidney and demonstrated that Atp6ap2 mediates HFD-induced renal gluconeogenesis via PGC1-α/AKT-1 pathway.

## Materials and methods

### Animals

All experimental protocols were approved by the University of Virginia (UVA) Animal Care and Use Committee (ACUC). All experiments were carried out in accordance with the UVA ACUC. The study was carried out in compliance with the ARRIVE guidelines. Experiments were conducted in 8-week-old male C57BL/6 (BL6) mice (Jackson Laboratory, USA) and inducible nephron specific Atp6ap2-knockout (KO) mice weighing 20–25 g. To induce nephron-wide Atp6ap2-KO, mice were treated with 2 mg/ml doxycycline in 2% sucrose drinking water for 12 days while control mice received 2% sucrose water only for the same period. Mice were provided tap water ad libitum and normal diet (ND, 12% fat, 0.4% sodium chloride Harlan-Teklad, Madison, WI) or high fat diet (HFD, 45% fat from lard, 0.3% sodium chloride, Research Diets, New Brunswick, NJ). Animals were allowed 1 week to adjust to our animal care facility. Animals were divided randomly into four groups: ND group (ND, n = 4–6), ND + KO (n = 5), HFD (n = 4–6), and HFD + KO (n = 4–6). All mice were given free access to normal tap water for the duration of the study.

### Protein extraction and Western blotting

For protein extraction, kidney cortex was lysed in T-PER buffer and a protease inhibitor cocktail (Thermo Scientific). Clear protein extracts were obtained by centrifugation at 12,000 rpm for 10 min at 4 °C. Protein concentrations were determined by BCA protein assay, and 20–40 µg of protein mixed with loading buffer was loaded per lane. Proteins were transferred to polyvinylidene difluoride (PVDF) membrane filters (Millipore). PVDF membranes were blocked with 5% dry milk for 1 h. Membranes were incubated in the primary antibody overnight at 4 °C. The following antibodies were used in Western blotting: Atp6ap2 (HPA 003156, 1:1000 sigma Aldrich, USA); PEPCK (1:1000; abcam, ab28455); FBPase (1:500 dilution; Sigma, HPA005857); G6Pase (1:200, Norvus biologicals, NBP180533); PGC-1α (1:200 dilution; abcam, ab54481); p-AKT (1:1000 cell signaling, 9271S); t-AKT (1:1000, cell signaling, 9272S). The membranes were then incubated with the corresponding secondary antibody (1:5000, horseradish peroxidase-conjugated anti-rabbit) in TBST-5% nonfat milk for 1 h at room temperature, and the immunoreactive bands were visualized by chemiluminescence methods and visualized followed by incubation with horseradish peroxidase-labeled IgG (1∶5000). The immunoreactive bands were detected by chemiluminescence methods and visualized on ChemiDoc Imaging system (Life Science Research, Bio Rad, CA, USA). Densitometric analysis of the images was performed using the Image J software (NIH, Bethesda, MD, USA). Protein expressions were normalized to β-actin protein (1:5000 dilutions, Santa Cruz, Dallas, TX, USA).

### Real-time PCR: determination of mRNA expression

Total RNA from tissues were extracted using TRIzol reagent (Invitrogen, Carlsbad, CA. USA) according to the manufacturer's protocol. RNA concentration was measured by NanoDrop 1000 (Thermo Fisher Scientific). Aliquots of total RNA (1 µg) from each sample were utilized and single-stranded cDNA was synthesized using iScript cDNA Synthesis Kit (Bio-Rad). PCR was performed with iQTM SYBR green supermix (Bio-Rad), as the fluorescence indicator, according to the manufacturer's instructions. Expression levels of Atp6ap2, PEPCK, G6PAse and FBPase mRNA were measured by a real-time RT-PCR iCycler according to the manufacturer's instructions (Bio-Rad, Hercules CA, USA). Primers sequences used in this study are as follows: Atp6ap2, forward 5′TGGCCTATACCAGGAGATCG 3′, reverse 5′-AATAGGTTGCCCACAGCAAG-3′; PEPCK, forward 5′-TGCGGATCATGACTCGGATG -3′, reverse 5′-AGGCCCAGTTGTTGACCAAA-3′, G6PAse forward 5′-TTTCCCCACCAGGTCGTGGCT -3′ reverse 5′-CCCATTCTGGCCGCTCACAC-3′, FBPase forward 5′-ATGGTATCGCTGGCTCAACC-3′ reverse 5′-ACAGGTAGCGTAGGACGACT-3′. Reactions were performed in triplicate, and threshold cycle numbers were averaged. The mRNA levels of target genes were normalized to the GAPDH mRNA levels. GAPDH forward 5′-ACCACAGTCCATGCCATCAC -3′ reverse 5′-TCCACCACCCTGTTGCTGTA-3′. Full blots are included in the supplementary file.

### Renal (pro)renin receptor immunostaining

Kidney tissue blocks were prepared, deparaffinized, and sectioned as described previously. Sections were incubated overnight at 4 °C with primary antibody directed against Atp6ap2 antibody (1:100 dilution; HPA 003156, Sigma, Cambridge, USA). A negative control (IgG) was included by omitting the primary antibody. The immunostaining images were captured by light microscopy using a Qimaging Micropublisher 5.0 RTV camera coupled to a Zeiss Axiophot microscopy (Carl Zeiss, Jena, Germany).

### Statistical analysis

Comparisons among different treatment groups were assessed by one-way ANOVA followed by a Tukey test for *post-hoc* comparisons. Data are expressed as mean ± SEM. *P* < 0.05 is considered statistically significant.

## Supplementary Information

Supplementary Figures.
