# Supplementary material for: Novel regulation of renal gluconeogenesis by Atp6ap2 in response to high fat diet via PGC1-α/AKT-1 pathway
Source: Sci Rep. 2021 May 31;11:11367. doi: 10.1038/s41598-021-90952-7 (PMC8167177; doi:10.1038/s41598-021-90952-7)
Supplement: Supplementary file 1 — Supplementary Figures. [file 41598_2021_90952_MOESM1_ESM.pdf]

Novel regulation of renal gluconeogenesis by Atp6ap2/(Pro)renin receptor in response to high fat diet via PGC1-α/AKT-1 pathway

Safia Akhtar, Silas A Culver and Helmy M Siragy*

Department of Medicine, University of Virginia, Virginia, USA.

***Abbreviated Title**:* Atp6ap2/**(**Pro)renin receptor upregulates renal gluconeogenesis in response to HFD

Key terms: Atp6ap2/**(**Pro)renin receptor, renal gluconeogenesis, high-fat diet

Corresponding author:

Helmy M. Siragy, MD

P.O. Box 801409, University of Virginia Health System, Charlottesville, VA 22903

Telephone: 434-924-5629

Fax: 434-982-3626

Email: hms7a@virginia.edu

FULL WESTERN BLOT

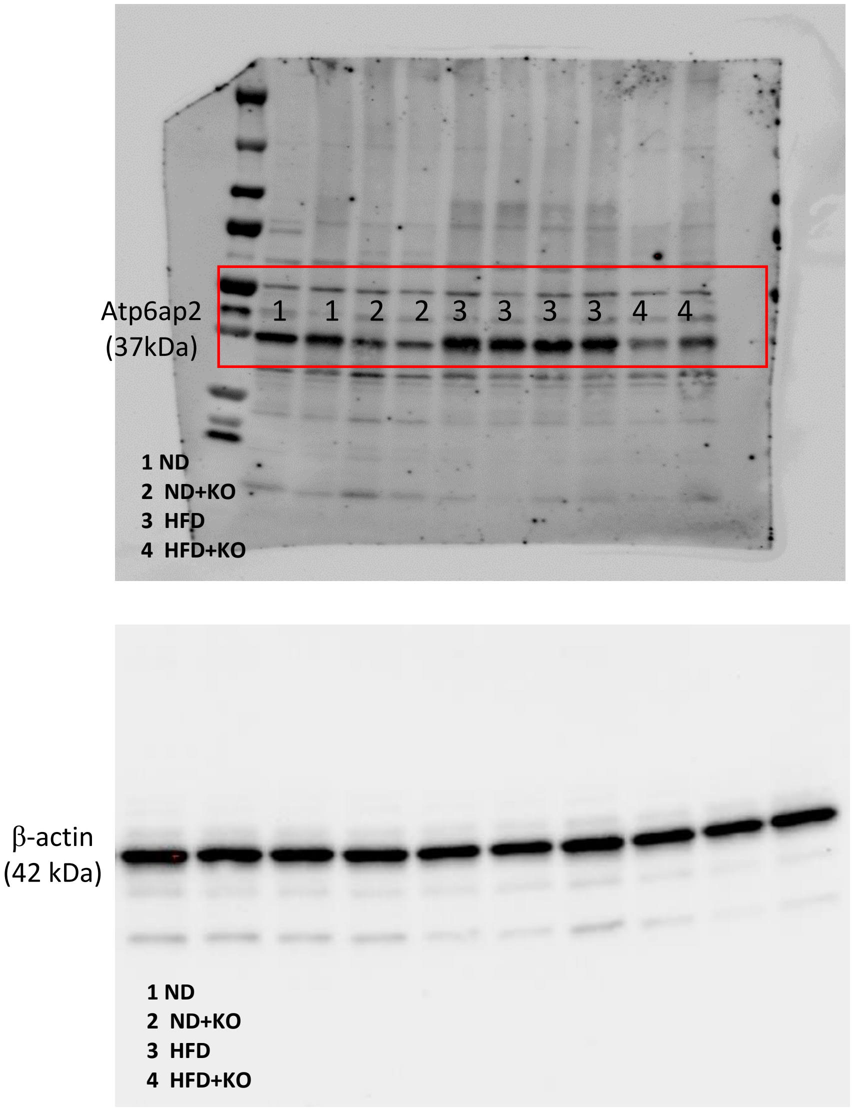

S1 Fig: Raw western blot image of renal cortical protein expressions of **Atp6ap2 and β-actin** protein expressions in 8 weeks following normal diet (ND) and high fat diet (HFD) in mice with or without Atp6ap2-KO (correspond to Fig 2B in the manuscript).

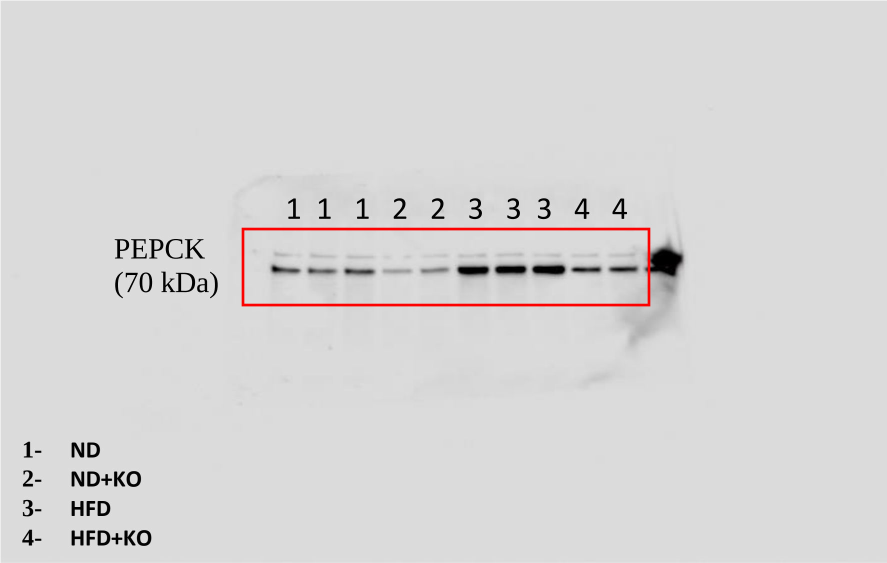

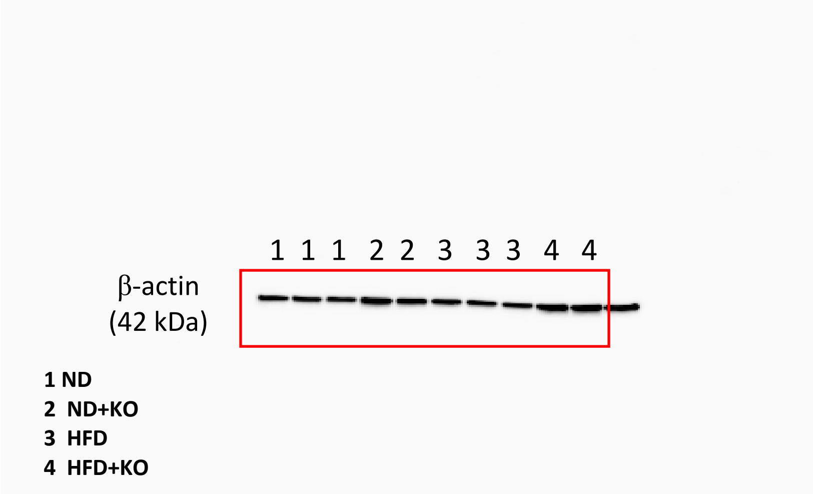

S2 Fig: Raw western blot image of renal cortical protein expressions of **PEPCK and β-actin** protein expressions in 8 weeks following normal diet (ND) and high fat diet (HFD) in mice with or without Atp6ap2-KO (correspond to Fig 4B in the manuscript).

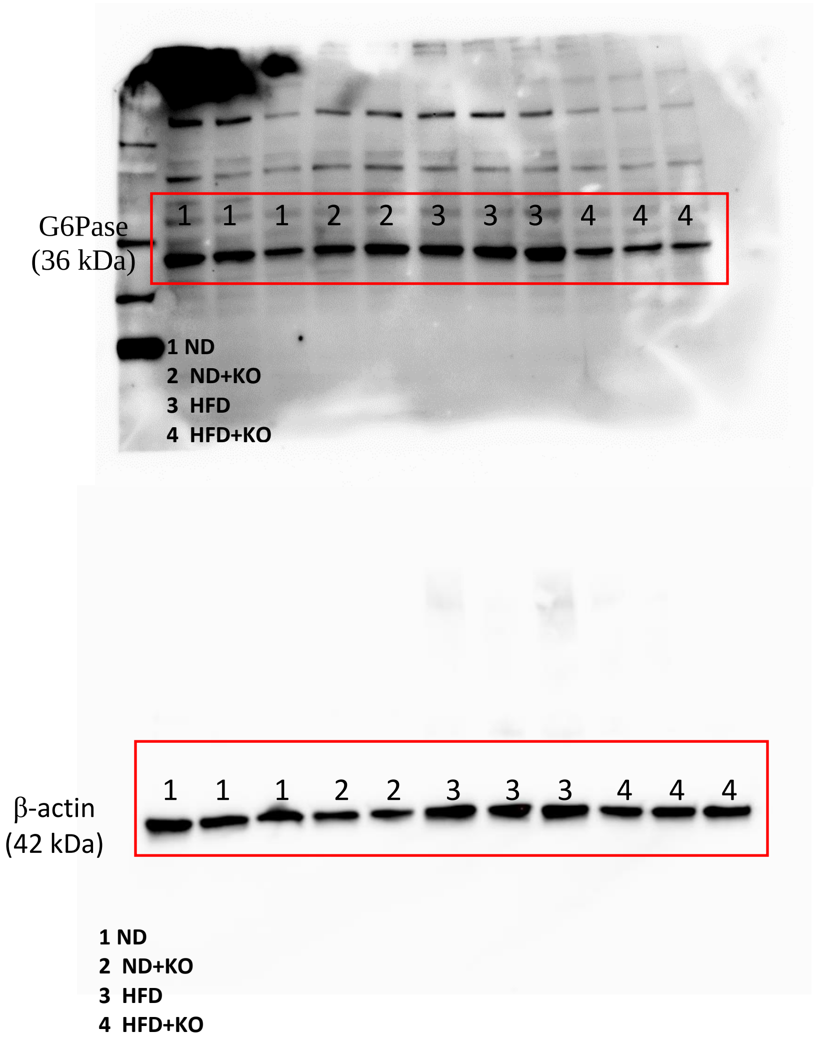

S3 Fig: Raw western blot image of renal cortical protein expressions of **G6PAse and β-actin** protein expressions in 8 weeks following normal diet (ND) and high fat diet (HFD) in mice with or without Atp6ap2-KO (correspond to Fig 5B in the manuscript).

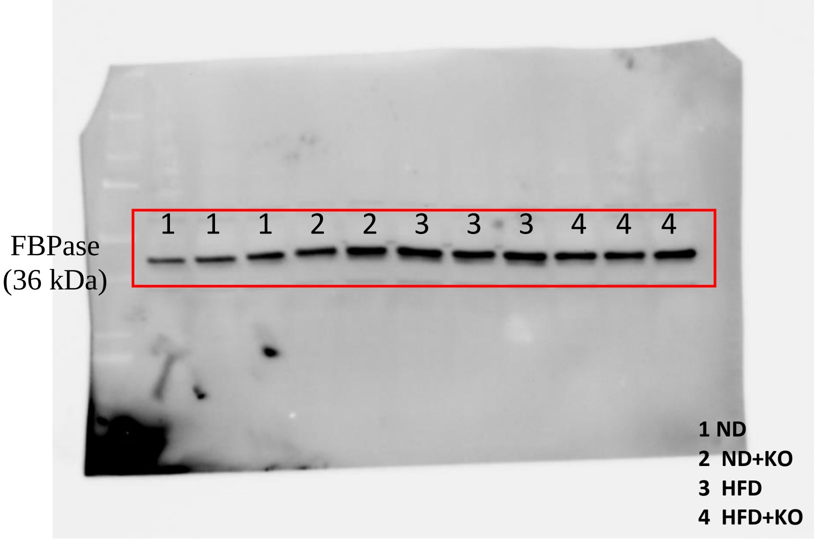

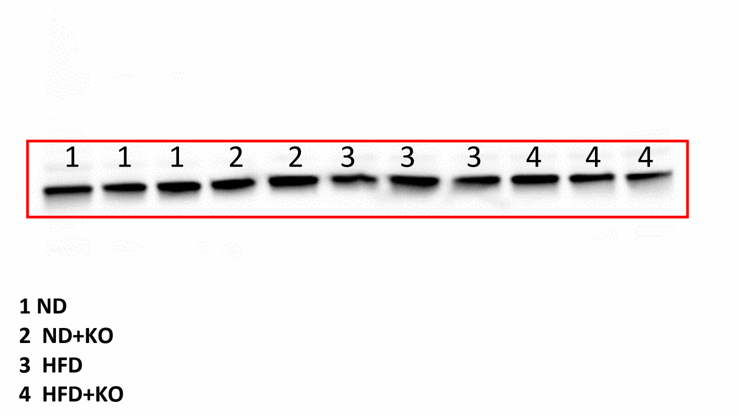

S4 Fig: Raw western blot image of renal cortical protein expressions of **FBPase and β-actin** protein expressions in 8 weeks following normal diet (ND) and high fat diet (HFD) in mice with or without Atp6ap2-KO (correspond to Fig 6B in the manuscript).

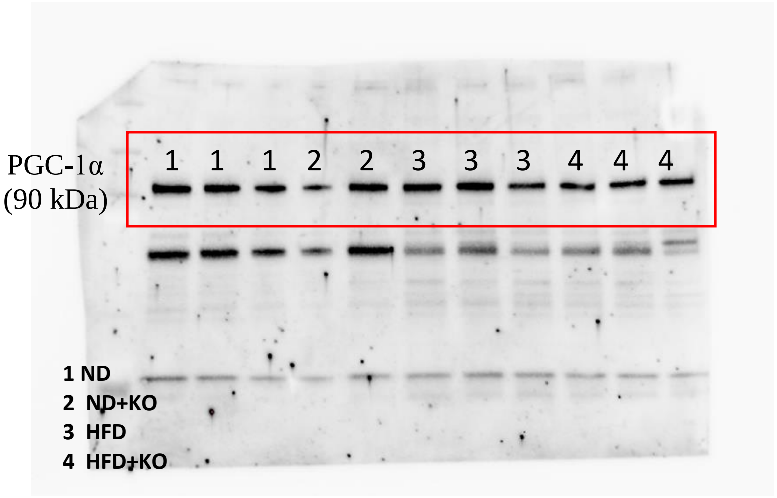

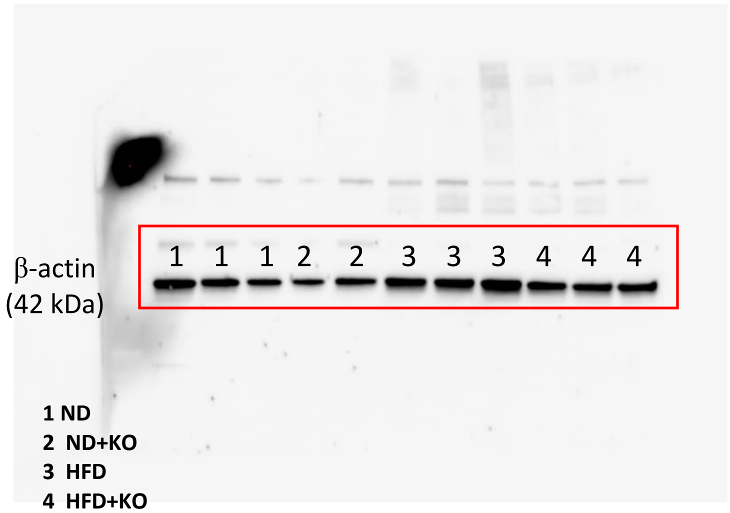

S5 Fig: Raw western blot image of renal cortical protein expressions of **PGC-1α and β-actin** protein expressions in 8 weeks following normal diet (ND) and high fat diet (HFD) in mice with or without Atp6ap2-KO (correspond to Fig 7A in the manuscript).

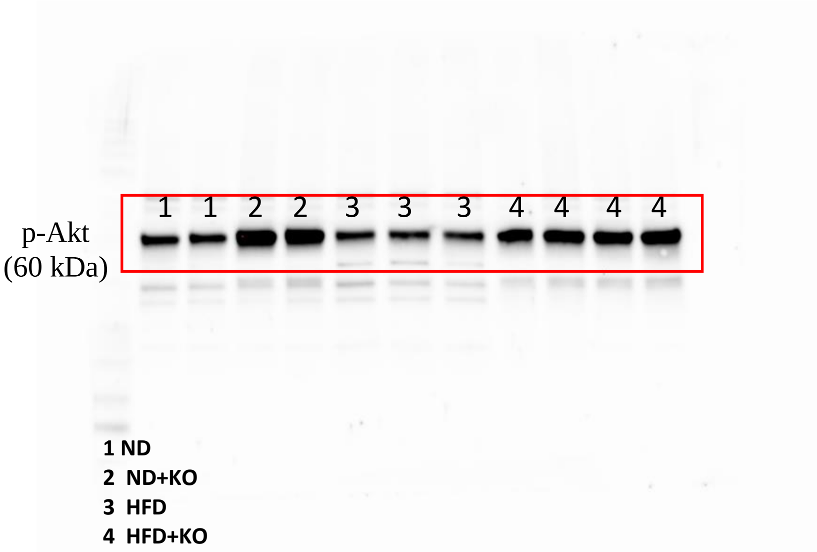

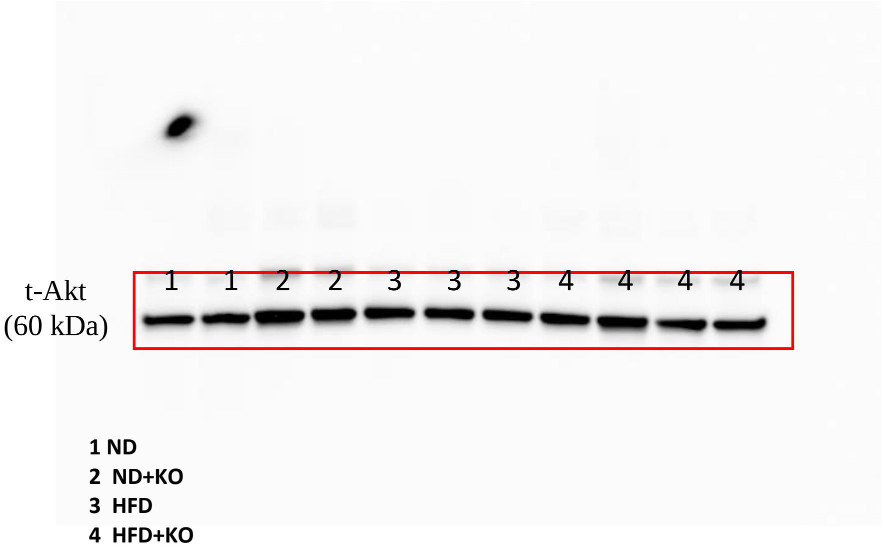

S6 Fig: Raw western blot image of renal cortical protein expressions of **p-Akt and t-Akt** protein expressions in 8 weeks following normal diet (ND) and high fat diet (HFD) in mice with or without Atp6ap2-KO (correspond to Fig 7B in the manuscript).
